# Biomechanical study of three kinds of internal fixation for the treatment of sacroiliac joint disruption using biomechanical test and finite element analysis

**DOI:** 10.1186/s13018-018-0858-2

**Published:** 2018-06-19

**Authors:** Tao Wu, Xuejiao Ren, Yunwei Cui, Xiaodong Cheng, Shuo Peng, Zhiyong Hou, Yongtai Han

**Affiliations:** 1 Department of Bone Disease, Third Hospital of Hebei Medical University, Shijiazhuang, 050051 Hebei China; 2 Department of Radiotherapy, Fourth Hospital of Hebei Medical University, Shijiazhuang, 050011 Hebei China; 3 Department of Trauma, Third Hospital of Hebei Medical University, Shijiazhuang, 050051 Hebei China

**Keywords:** Biomechanics, Internal fixation, Sacroiliac joint disruption, Finite element analyses

## Abstract

**Background:**

To compare the stability of sacroiliac joint disruption fixed with three kinds of internal fixation using both biomechanical test and finite element analysis.

**Methods:**

Five embalmed specimens of an adult were used. The symphysis pubis rupture and left sacroiliac joint disruption were created. The symphysis pubis was stabilized with a five-hole plate. The sacroiliac joint disruption was fixed with three kinds of internal fixation in a randomized design. Displacements of the whole specimen and shifts in the gap were recorded. Three-dimensional finite element models of the pelvis, the pelvis with symphysis pubis rupture and left sacroiliac joint disruption, and three kinds of internal fixation techniques were created and analyzed.

**Results:**

Under the vertical load, the displacements and shifts in the gap of the pelvis fixed with minimally invasive adjustable plate (MIAP) combined with one iliosacral (IS) screw were the smallest, and the average displacements of the pelvis fixed with an anterior plate were the largest one. The differences among them were significant. In finite element analysis and MIAP combined with one IS screw fixation showed relatively best fixation stability and lowest risks of implant failure than two IS screws fixation and anterior plate fixation.

**Conclusion:**

The stability of sacroiliac joint disruption fixed with MIAP combined with one IS screw is better than that fixed with two IS screws and anterior plate under vertical load.

## Background

Sacroiliac joints (SIJs) play an important role in the pelvic ring, which is the main structure of force transmitting between the upper and lower limbs [1]. Sacroiliac joint disruption (SJD) is a severe clinical injury, caused by high-energy traumas. Although surgical treatment has become a gold-standard method for SJD in the recent years, how to select an appropriate fixation technique remains a challenging problem for clinical surgeon [2].

Currently, there are several internal fixation techniques for SJD, including percutaneous iliosacral (IS) screw, anterior plate, posterior transiliac plating, minimally invasive adjustable plate (MIAP), and so on [3–5]. These fixations have several advantages and disadvantages respectively, and none is proved to be the strongest fixation in the experiments and clinics.

The percutaneous IS screws are widely used due to its advantage of a minimal incision at present. Osterhoff et al. treated the patients with unstable pelvic fracture using IS screws and found that IS screw fixation was a sufficient technique [6]. However, this fixation requires extensive experience and has a high rate of iatrogenic vascular and neural injuries. Both patients and doctors are exposed to lots of radiation during IS screw placement [7]. The anterior plate is another therapeutic method, which can also provide biomechanical stability of the SIJs. Simpson et al. found that satisfactory clinical results were achieved by using anterior plate fixation. However, screw loosening occurred during the follow-up [8]. To avoid these limitations, Chen et al. introduced MIAP [9], which simulates the structures of the sacroiliac joint complex. It could obtain a satisfactory result when MIAP was used for treating unstable pelvic ring injuries [5, 9]. In biomechanical test, the stability of sacral fracture fixed with MIAP was inferior than that fixed with two IS screws; however, these differences were not significant [10].

Clinically, surgeons try to find the strongest internal fixation for SJD. Stable SIJs can reduce the risk of low back pain postoperation and ask the patients to walk earlier which could avoid lots of long-term complications caused by bed rest. Therefore, we hypothesize that SJD fixed with MIAP combined with one IS screw is more stable than that with an anterior plate and two IS screws. It is well known that it is very difficult to compare the stability of different fixations for SJD in clinical applications because of the variations in fracture patterns, bone quality, and fixation. So, biomechanical test and three-dimensional finite element analysis (FEA) are the most commonly used methods in orthopedic biomechanical research. In this study, we aimed to compare the stability of SJD fixed with three kinds of internal fixation using both biomechanical test and finite element analysis and provide a basis for the clinical application.

## Methods

### Structure of minimally invasive adjustable plate

**Fig. 1:**
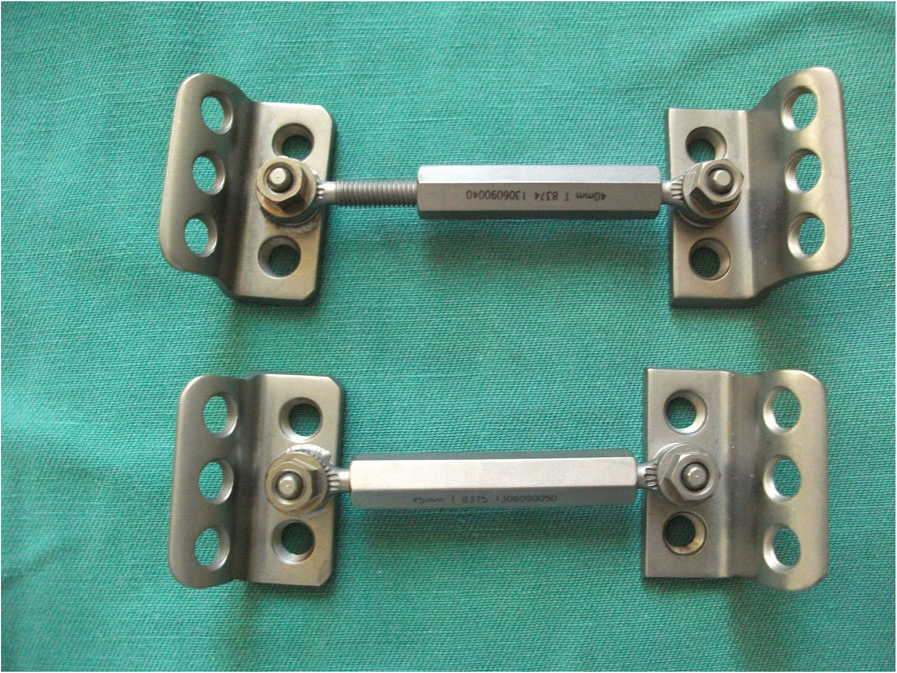
The structure of MIAP

MIAP is made up of three parts: two similar Z-shaped brackets and an adjustable connection bar (Fig. 1). Each similar Z-shaped bracket is composed of a lower wing and a cambered wing. The lower wing of this bracket is placed on the dorsal surface of the sacrum, and the cambered wing is positioned close to the dorsal surface of the posterior superior iliac spines, which is fixed to the sacrum and ilium using some long cancellous screws.

### Preparation and preservation of specimens

Five embalmed adult male cadaver pelvises (age 43.2 ± 6.9 years) were used for the biomechanical test, which were provided by the Department of Anatomy of Hebei Medical University. The inclusion criteria for pelvic specimens were as follows: (1) The hip joint and pubic symphysis must be intact. (2) The soft tissues of the specimens were removed, and the main ligaments were left intact. (3) The specimens from patients with rheumatism, tuberculosis, anatomic variations, cancer, and other diseases were excluded. Specimens that were proven to have osteoporosis using an osteocore 3 dual energy X-ray osteodensitometer (Medilink Company, Parc de la Mediterranee, France) were excluded (Table 1). These specimens were stored at − 20 °C and melted at room temperature 12 h before the test.

**Table 1:** Specimen information and sequence of internal fixation

Sequence number	Age (years)	Bone mineral density (*T* score)	Sequence of fixation
1	45	0.3	①-②-③
2	35	0.4	②-③-①
3	51	0.2	③-①-②
4	48	0.2	①-③-②
5	37	0.5	②-①-③

① MIAP combined with one IS screw; ② two IS screws; ③ anterior plate

### Modeling sacroiliac joint disruption and fixation of specimens

**Fig. 2:**
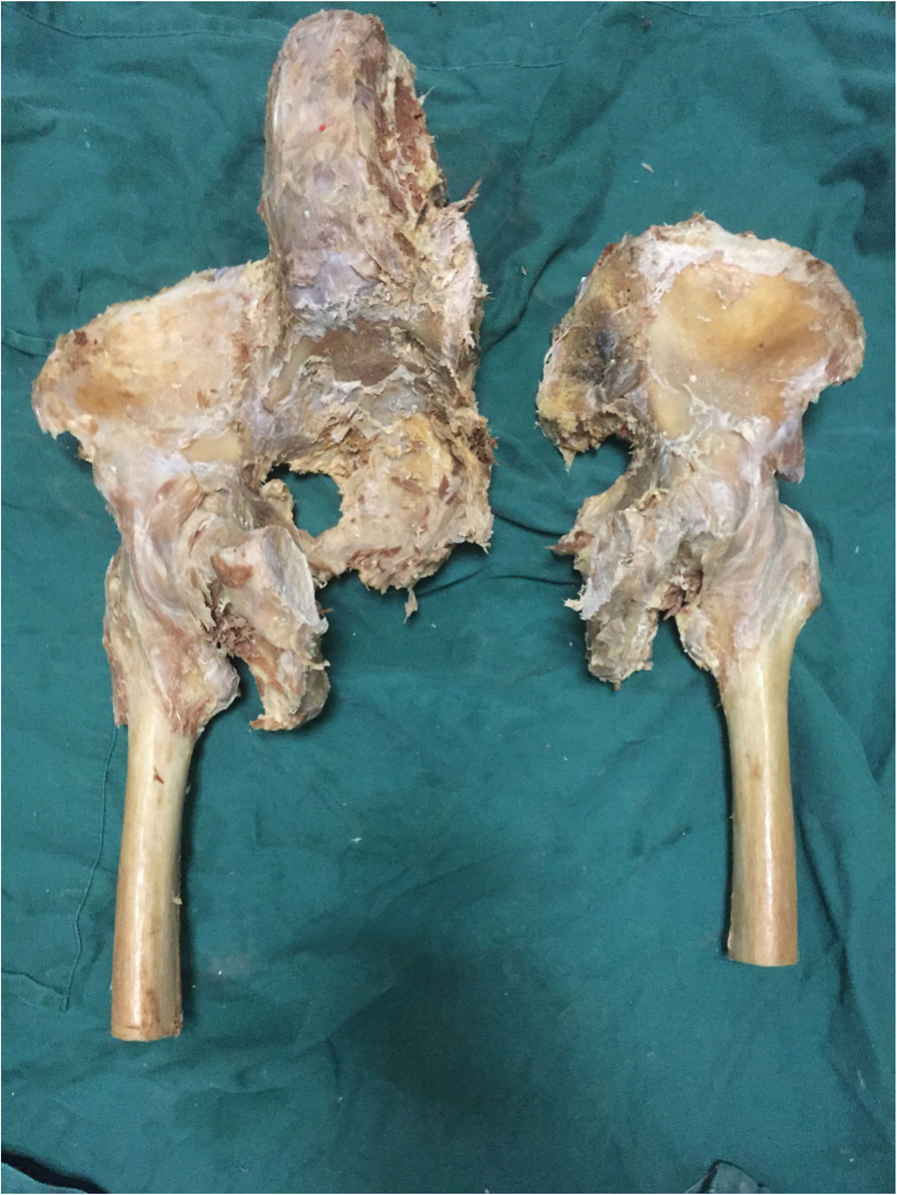
Image of the specimen after creating left sacroiliac disruption with an incision on the symphysis pubis

For better comparing the stability of internal fixation, the anterior and posterior pelvic rings were disrupted (Fig. 2). Anteriorly, a symphysis pubis rupture was made, which was stabilized using a five-hole plate. Posteriorly, the disruption of sacroiliac joint was manipulated by cutting the connection between the left sacroiliac joint. Three types of internal fixation were implanted randomly after the reduction of SJD (Table 1).

**Fig. 3:**
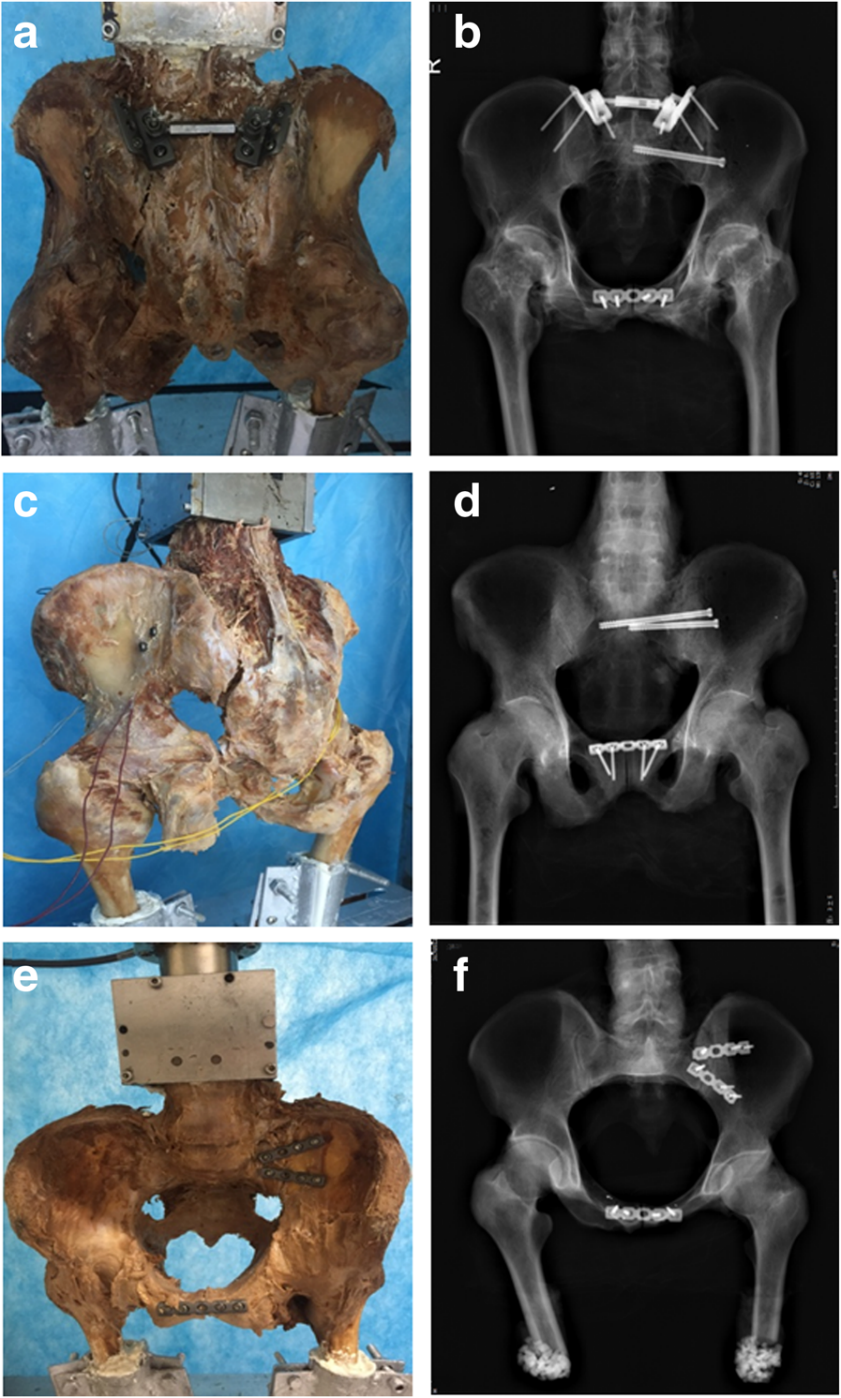
Sacroiliac disruption fixed with MIAP combined with one IS screw. **a** Posterior view of the specimen. **b** Anteroposterior radiograph of the pelvis. Sacroiliac disruption fixed with two IS screws. **c** Posterior view of the specimen. **d** Anteroposterior radiograph of the pelvis. Sacroiliac disruption fixed with an anterior plate. **e**, **a** Posterior view of the specimen. **f**, **b** Anteroposterior radiograph of the pelvis

Three kinds of internal fixation were used as follows:

1. MIAP combined with one IS screw group: To expose the posterior side of the ilium and sacrum, choose an appropriate MIAP. Some appropriate long cancellous screws were implanted into the ilium and sacrum respectively. Afterwards, one 2.0-mm Kirschner wire was inserted through the ipsilateral external surface of the ilium and into the first sacral vertebral body. Fluoroscopy was used to confirm appropriate screw position. One 7.3-mm cannulated, partially threaded, and cancellous IS screw was inserted into the first sacral vertebral body along the Kirschner wire (Fig. 3).
2. Two IS screws group: Two 2.0-mm Kirschner wires were inserted into the first sacral vertebral body according to the above method. Fluoroscopy was also used to confirm the screws’ positions. And then, two appropriate 7.3-mm cannulated, partially threaded, and cancellous IS screws were inserted along these wires simultaneously (Fig. 3).
3. Anterior plate group: The anterior plate was bent according to the anterior structure of SIJs. Appropriate screws were inserted into the sacrum and ilium anteriorly (Fig. 3).

### Measurements

The L4 vertebral body and bilateral distal femurs of the specimens were fastened in the Electroforce 3520-AT Bose biomechanical testing machine (BOSE Corporation, Eden Prairie, USA) at a neutral position. The pins of a grating displacement sensor (Guangzhou Lokshun CNC Equipment Ltd., Guangzhou, China) were set to the level of the posterior inferior iliac spine (PIIS), which was fastened to the testing machine. A vertical load of 200 N was applied to eliminate creep after implanting each internal fixation. The vertical cyclic load was between 0 and 500 N and increased at a rate of 10 N/s. The cyclic load was applied in 30 cycles. In the last three cycles, the displacement of the specimen was recorded at vertical loads of 100, 200, 300, 400, and 500 N by the machine. Shifts between the gap were measured at a vertical load of 500 N.

### Statistical analysis

The statistical analyses were performed using SPSS version 16.0 software (SPSS Inc., Chicago, IL, USA). The data were presented as mean ± SD. Model displacement and shifts between the gap were compared by randomized block design ANOVA. The Bonferroni test was used to compare significant differences between the groups. Significance was set at *P* < 0.05.

### Finite element models and implants

A three-dimensional finite element model was created from CT scan. The intact pelvis was scanned from an adult healthy volunteer, and the DICOM format files were processed by MIMICS 10.01 (Materialise, Belgium). And then, these files were processed by Geomagic Studio 12 (Geomagic, USA) and Abaqus 6.11-1 (SIMULIA, France). The full pelvis was composed of the left ilium, sacrum, right ilium, and symphysis pubis, and these bones consisted of the cortical bone and cancellous bone. The anterior sacroiliac, interosseous sacroiliac, posterior sacroiliac, sacrotuberous, and sacrospinous ligaments were also created to simulate normal condition. The linear elastic isotropic material properties were used, and the properties of the bones and ligaments are shown in Table 2. The sacroiliac joint and symphysis pubis were modeled with contact type “bonded.” In the finite element models, the bilateral acetabulums were fully fixed and a vertical load of 500 N was applied on the upper surface of the sacrum, which was equal to the upper body weight.

**Table 2:** The properties of materials used in pelvic finite element model

Material	Young’s modulus (MPa)	Poisson’s ratio *u*	*K* (N/mm)
Cortical bone (ilium)	17,000	0.3	
Cortical bone (sacrum)	6140	0.3	
Cancellous bone (ilium)	132	0.2	
Cancellous bone (sacrum)	1400	0.3	
Symphysis pubis	5	0.45	
Sacroiliac posterior long ligament			1000
Sacroiliac posterior short ligament			400
Sacroiliac anterior ligament			700
Sacrotuberous ligament			1500
Sacrospinous ligament			1400

Three kinds of internal fixation model, which included percutaneous IS screws, anterior plate, and MIAP, were created by UG (Unigraphics NX) software according to their structural features, and the threads of screws were omitted so as to simplify the models.

### Injured model and finite element analyses

The connection of the left symphysis pubis and left sacroiliac joint was deleted, and the left anterior sacroiliac, interosseous sacroiliac, posterior sacroiliac, sacrotuberous, and sacrospinous ligaments of the pelvic model were also deleted, which were consistent with the injured specimen of the biomechanical test.

The injured model was fixed by MIAP combined with one IS screw, two IS screws, and anterior plate in sequence. The vertical force and boundary conditions were the same in different models. In post-processing, the von Mises of the pelvis, the maximum displacement of the whole pelvis, the maximum von Mises stress of internal fixation, and the shifts of the gap at the level of PIIS were calculated to compare the different kinds of internal fixation.

## Results

### Biomechanical test

**Fig. 4:**
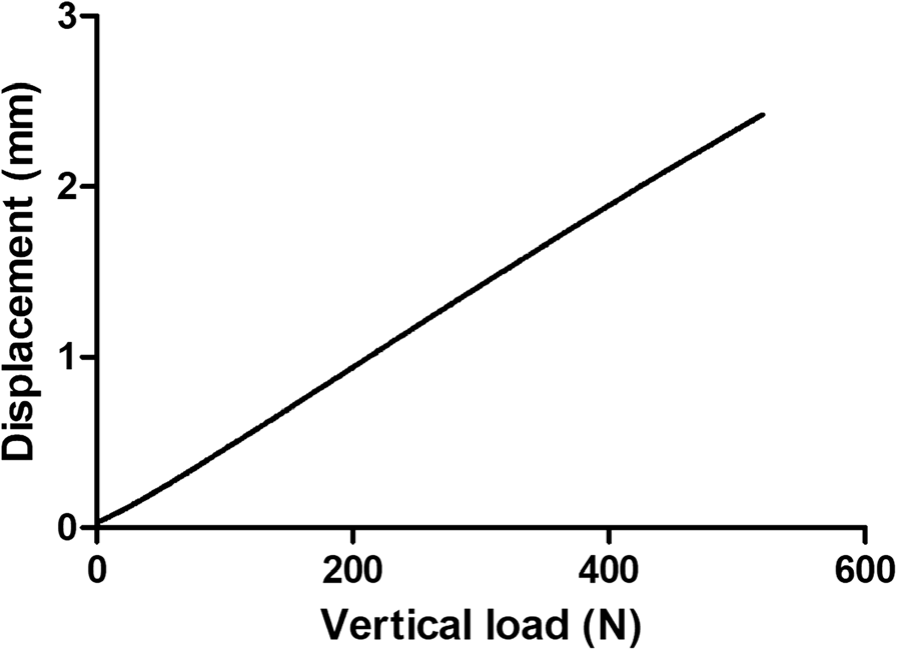
Load-displacement scattergraph of the specimens

All specimens were fastened at a neutral position without obvious fracture or obliquity. Evulsion, loosening, and breakage of internal fixation were not observed. The displacements of specimens were recorded by BOSE biomechanical workstation under vertical load. The shifts between the gap were recorded simultaneously. Based on load-displacement scattergraph, the smooth straight line indicated that the specimens had elastic deformation (Fig. 4).

Under different vertical load, the average displacements of the pelvis fixed with MIAP combined with one IS screw were the smallest, and the average displacements of the pelvis fixed with an anterior plate were the largest one. The value of the pelvis fixed with two IS screws was in the middle. The differences among them were significant (Table 3).

**Table 3:** Displacement of the pelvis fixed with three types of internal fixation under vertical load (‾*x* ± *s*, *n* = 5)

Load (N)	① (mm)	② (mm)	③ (mm)	*F* value/*p* value	*p* value		
					① vs ②	① vs ③	② vs ③
100	0.496 ± 0.102	0.6768 ± 0.130	1.1826 ± 0.220	78.209/0.000	0.039	0.000	0.000
200	0.945 ± 0.193	1.334 ± 0.272	2.002 ± 0.296	87.893/0.000	0.004	0.000	0.000
300	1.466 ± 0.311	1.956 ± 0.342	2.832 ± 0.366	203.61/0.000	0.000	0.000	0.000
400	1.865 ± 0.369	2.478 ± 0.392	3.871 ± 0.601	207.281/0.000	0.001	0.000	0.000
500	2.477 ± 0.321	3.128 ± 0.519	4.704 ± 0.600	129.958/0.000	0.005	0.000	0.000

① MIAP combined with one IS screw; ② two IS screws; ③ anterior plate

Under vertical load of 500 N, the average shifts in the SIJ gap of the pelvis fixed with MIAP combined with one IS screw were significantly the smallest one[0.619 ± 0.117 mm], followed by that of fixed with two IS screws [0.893 ± 0.236 mm], and the largest one was that of fixed with anterior plate [1.747. ± 0.192 mm]. The differences were also significant (*P* < 0.01).

### Finite element analyses

**Fig. 5:**
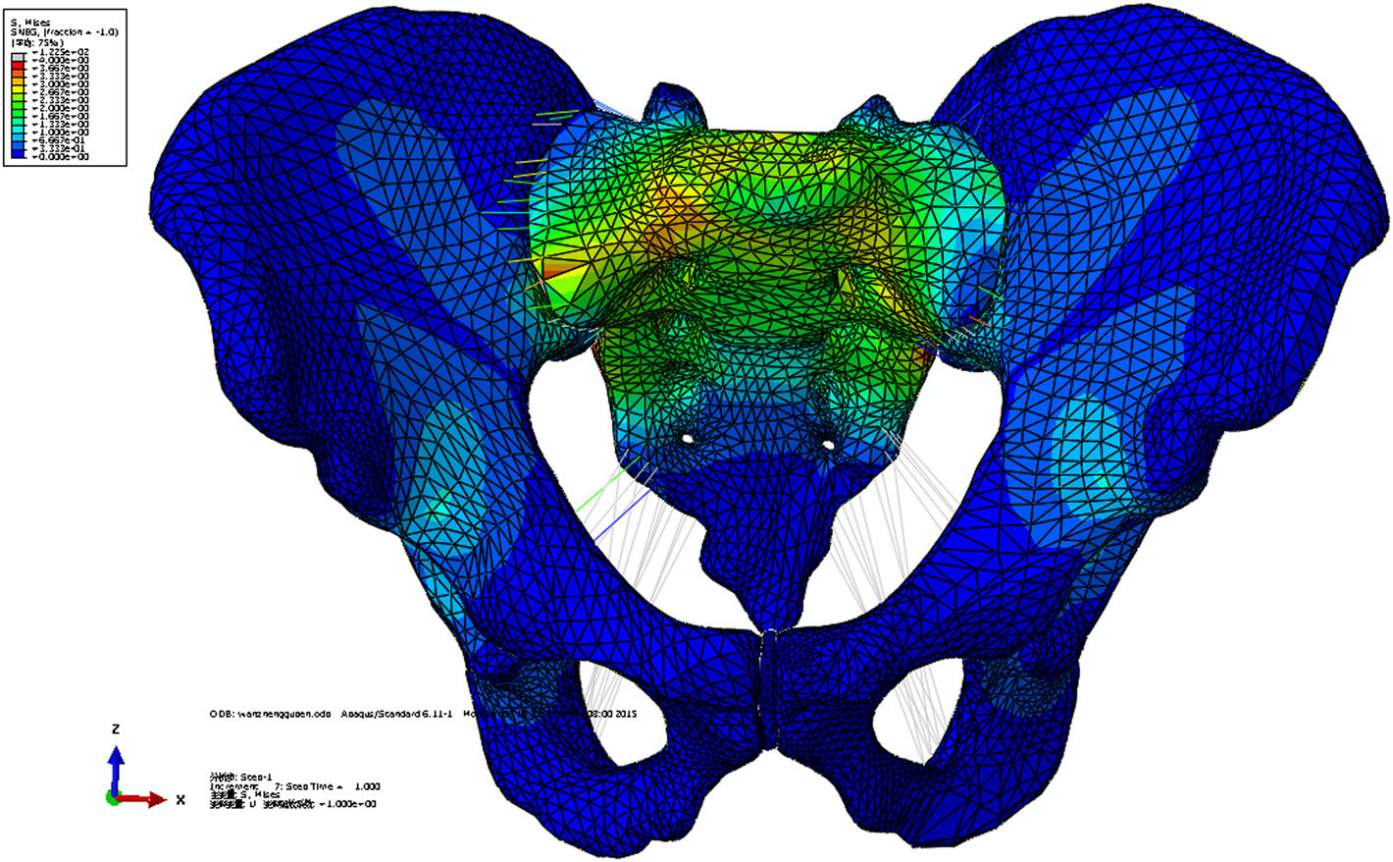
Distribution of von Mises stress in the intact pelvic model

Under a vertical load of 500 N, the distribution of von Mises stresses in intact pelvic model showed that the pathway of the vertical load was from the upper surface of the sacrum, through the bilateral sacral wing, sacroiliac joint, large sciatic notch, and iliac arcuate line, to the bilateral acetabulum (Fig. 5). The maximum von Mises stresses located at the large sciatic notch and the von Mises stresses of the anterior pelvic ring were small.

**Fig. 6:**
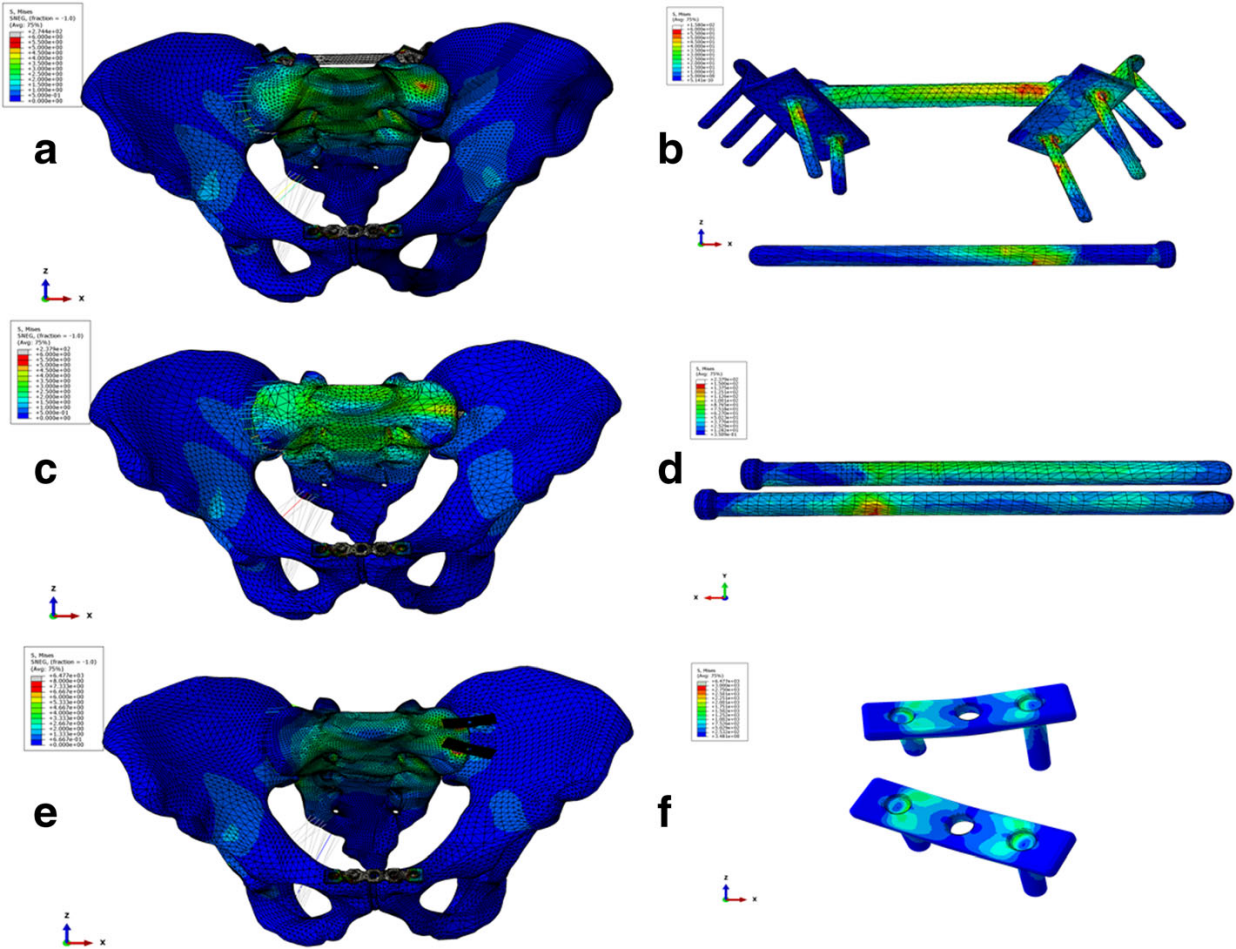
**a** The stress distribution of the pelvis fixed with MIAP combined with one IS screw. **b** The stress distribution of the MIAP combined with one IS screw. **c** The stress distribution of the pelvis fixed with two IS screws. **d** The stress distribution of two IS screws. **e** The stress distribution of the pelvis fixed with anterior plate. **f** The stress distribution of the anterior plate

Under a vertical load of 500 N, all treated models indicated that the distribution of stresses had been greatly restored, especially for MIAP combined with one IS screw fixation and two IS screws fixation (Fig. 6). The maximum displacement of the model fixed with MIAP combined with one IS screw, two IS screws, and anterior plate was 1.265, 1.377, and 2.223 mm, respectively. The higher maximum von Mises stress reveals that the model has a higher risk of broken implant. The maximum von Mises stress of MIAP combined with one IS screw was 374.44 MPa, located at the junction between the nail and plate of MIAP. The maximum von Mises stress of two IS screws was 513.64 MPa, located at the inferior IS screw. The maximum von Mises stress of anterior plate was 2476.57 MPa, located at the junction between the nail and plate of the inferior anterior plate (Fig. 6). The shifts at the gap of the model fixed with MIAP combined with one IS screw, two IS screws, and anterior plate were 0.746, 0.897, and 1.571 mm, respectively.

## Discussion

Biomechanical experiment and finite element analysis are the most commonly used methods in orthopedic biomechanical research. Biomechanical experiment is a traditional research method, which can be analyzed intuitively. However, the sources of specimens are fewer, and the qualities of the bones are different, which limit its application. FEA is a new research method in recent years. It simulates the physical condition of the bones and can carry out microcosmic mechanical research. FEA and biomechanical experiment can complement each other. In our study, we made the same injured pelvic model and the same internal fixation in biomechanical experiment and FEA, so as to verify their effects.

The smaller maximum displacement of the pelvis and shifts at the gap represent better fixation stability [11]. Our results showed that the stability of the pelvic ring fixed with an anterior plate was lower than that fixed with two IS screws and MIAP combined with one IS screw, which was consistent with the literature [12]. Although anterior approach can provide a direct visualization without lots of radiation exposure, and obtain good results in clinical practices [4], biomechanical research revealed that fixation by anterior approach was insufficient to maintain the stability of vertical injured pelvic ring [12]. The reason may be that anterior plate did not take into account the important role of the sacroiliac complex in the posterior pelvic ring, which only fixed the front of the sacroiliac joint, and the plate could be prebent according to the shape of the sacroiliac joint during implantation, which could damage the thread and reduce the strength of the plate. In addition, highly frequent complications represent after using an anterior plate, including the injury of the lumbosacral trunk and lateral femoral cutaneous nerve, back pain, minor claudication, and sexual dysfunction [13]. The high rate of screw loosening was another important factor restricting its application [4].

Percutaneous IS screw fixation is a common method applied for stabilizing the SJD. Two IS screws, inserted into the first sacral vertebral body, were regarded as the strongest fixation in stabilizing the posterior pelvic ring [14–16]. The IS screw penetrated through three layers of the cortical bone [17, 18], which was considered as “central fixation” technique. Although two IS screws fixation has some advantages including less blood loss, short incisions, and low soft tissue complications, it remains a technically difficult operation due to the limitations of clinical experience, medical facilities, and variations of anatomical structure. Recently, computer navigation is a useful technique for IS screw insertion that can reduce the malposition rate and operation time [19], but its widespread application was limited because of the demand of the expensive equipment and huge facilities. Under C-arm fluoroscopy, commonly used in operation room, it is very difficult to identify the “safe zone.” The incidence of sacral dysmorphism in adults is about 30–40% [20], so the safe zone of the upper sacrum is 36% smaller than normal [21]. It is reported that the screw misplacement rate was 2–13% due to malreduced or skeletal deformity [22].

It is difficult to insert two IS screws into the first sacral vertebral body. However, routine placement of single IS screw is safe, which was considered sufficient for stabilizing the posterior pelvic ring [23]. And the placement of two IS screws was shown to be clinically unreliable [24]. MIAP is a novel device for stabilizing the posterior pelvic ring. It is easy to perform without prolonging operation time and radiation exposure. The MIAP simulates the structure of the sacroiliac complex and is functioned as a suspension bridge. Biomechanical studies indicated that the stability of sacral fracture fixed with MIAP was inferior to that fixed with the two IS screws, but the difference was not significant [10]. We proposed that MIAP combined with one IS screw fixation, considered as “central and rear fixation” technique, should provide stronger fixation than two IS screws. In our study, both biomechanical test and FEA showed that the smaller maximum displacement of the pelvis and shifts at the gap were presented in the model fixed with MIAP combined with one IS screw than that fixed with two IS screws.

The injury of the sacroiliac joint is a source of low back pain, accounted for 15–30% of patients with chronic low back pain [25]. If the stresses on the injured side of the sacroiliac joint increased, degeneration may occur. Therefore, the best choice of internal fixation is to restore the distribution of stresses. The results of FEA in this study revealed that the distribution of stress in the pelvic model fixed with MIAP combined with one IS screw was most similar to that in the normal pelvic model, which has the lowest risk of low back pain. The lower maximum von Mises stress of fixation device shows a lower risk of internal fixation failure. The MIAP combined with one IS screw had the lowest maximum von Mises stress in these three kinds of internal fixation, which indicated that it is the best choice for stabilizing the posterior pelvic ring.

This study had several limitations. First, the biomechanical test had a small sample size. The power analysis of this study showed only 44.3% power to detect a significant difference at the *P* < 0.05 level, and it also showed that a large sample size (more than 12 specimens) would be required to detect any difference with 80% power. Artificial pelvises should be used in the biomechanical test in the future. Second, we implanted three kinds of internal fixation into the same specimen in sequence for reducing the influence caused by individual difference of specimens. But the use of pre-fixation could affect the holding power of the subsequent fixation. Therefore, we accurately located the direction and position of the screws using X-ray, in order to minimize the influence of screw channels. Third, we used the same IS screw between the MIAP combined with one IS group and two IS screws group, which could be slight loosening around previous IS screw during the biomechanical test. Fourth, only anterior plate on the sacroiliac joint was weak for vertically unstable pelvic ring injury. Future research should add an experimental group for comparing the stability of sacroiliac joint, which is anterior plate combined with one IS screw. Fifth, the parts of the bone were created as a linear elastic isotropic material in the FEA. The real structure of the bone is complex and different from that of FEA. And the FEA model did not contain the lumbar, femur, and muscle tissues. In future research, we should create a finite element model that is more approximate to the real bone structure.

## Conclusion

Three kinds of internal fixation are all useful for the treatment of SJD. The stability of sacroiliac joint disruption fixed with MIAP combined with one IS screw is better than that fixed with two IS screws and anterior plate under vertical load. We believe that this new technique is an effective surgical procedure.

## Abbreviations

CT: Computed tomography
DICOM: Digital imaging and communications of medicine
FEA: Finite element analysis
IS: Iliosacral
MIAP: Minimally invasive adjustable plate
PIIS: Posterior inferior iliac spine
SIJs: Sacroiliac joints
SJD: Sacroiliac joint disruption
